# Enhanced Pseudocapacitive Performance of Symmetric Polypyrrole-MnO_2_ Electrode and Polymer Gel Electrolyte

**DOI:** 10.3390/polym13203577

**Published:** 2021-10-16

**Authors:** Wen-Jun Zhuo, Yen-Hua Wang, Chia-Tse Huang, Ming-Jay Deng

**Affiliations:** Department of Applied Chemistry, Providence University, Taichung City 43301, Taiwan; s1070508@gm.pu.edu.tw (W.-J.Z.); s1070469@gm.pu.edu.tw (Y.-H.W.); kabanchan0630@gmail.com (C.-T.H.)

**Keywords:** flexible, solid-state supercapacitor, polypyrrole, MnO_2_, polymer gel electrolyte

## Abstract

Herein, the nanostructured polypyrrole-coated MnO_2_ nanofibers growth on carbon cloth (PPy-MnO_2_-CC) to serve as the electrodes used in conjunction with a quasi-ionic liquid-based polymer gel electrolyte (urea-LiClO_4_-PVA) for solid-state symmetric supercapacitors (SSCs). The resultant PPy-MnO_2_-CC solid-state SSCs exhibited a high specific capacitance of 270 F/g at 1.0 A/g in a stable and wide potential window of 2.1 V with a high energy/power density (165.3 Wh/kg at 1.0 kW/kg and 21.0 kW/kg at 86.4 Wh/kg) along with great cycling stability (capacitance retention of 92.1% retention after 3000 cycles) and rate capability (141 F/g at 20 A/g), exceeding most of the previously reported SSCs. The outstanding performance of the studied 2.1 V PPy-MnO_2_-CC flexible SSCs could be attributed to the nanostructured PPy-coated MnO_2_ composite electrode and the urea-LiClO_4_-PVA polymer gel electrolyte design. In addition, the PPy-MnO_2_-CC solid-state SSCs could effectively retain their electrochemical performance at various bending angles, demonstrating their huge potential as power sources for flexible and lightweight electronic devices. This work offers an easy way to design and achieve light weight and high-performance SSCs with enhanced energy/power density.

## 1. Introduction

Wearable, conformable electronic devices have become increasingly popular as individuals continue to actively pursue more convenient, safer, and comfortable lifestyles [1,2]. Here, lightweight, wearable, portable, and small-scale energy storage systems are required for use in conjunction with flexible/wearable electronic devices [3,4,5]. As part of a new form of eco-friendly high-performance energy storage systems, supercapacitors (SCs) have received a great deal of attention globally owing to their long cycle life, ultrafast charge/discharge rates, and higher power density compared to batteries [4,5,6]. However, the commercialization of SCs remains seriously hindered by their poor energy density compared to that of batteries [7,8,9]. The energy density *(E)* can be estimated using the equation *E* = 1/2 *CV*^2^, meaning an improvement in energy density can be attained by enhancing the operating voltage (*V*) or specific capacitance (*C*) of the energy storage systems [10,11,12]. In fact, because of the limitation of water splitting, the operating voltage of SCs is generally lower than 1.23 V in aqueous electrolyte systems [12,13]. Wang et al. constructed a 3.5 V solid-state fiber SC using an ionic liquid-based electrolyte combined with a polymer gel electrolyte, with the augmented solid-state SC providing high volumetric energy densities [14]. The same authors also devised an all-in-one fiber sensing device by combining a power-supporting SC with strain investigation [15]. In view of this, constructing high-pseudocapacitive-performance SCs is seen as a prospective option for increasing the operating voltage through controlling the electrodes and electrolytes. 

Here, a simple strategy involves constructing ionic liquid-based polymer gel electrolyte symmetric SCs (SSCs), which can both provide enhanced energy density and operate in a wide voltage range [16,17,18]. This type of high-performance ionic liquid-based polymer gel electrolyte SC has huge potential for application in flexible and portable electronic devices. 

In addition to the ionic liquid-based polymer gel electrolyte, the electrochemical properties of SCs are strongly associated with the active materials of the electrodes (cathode and anode). As such, great efforts have been made to investigate commonly applicable and high-capacity materials for use as electrodes for high-performance SCs. Among the previously reported active materials, MnO_2_ has been commonly used because of its environmentally friendly nature, low cost, large theoretical specific capacitance (1375 F/g), and high positive operating voltage, which are significant for SC electrode application. However, the small loading weight and low stability of such active materials have limited their use in real SC devices [19,20,21,22,23,24,25]. Recently, a number of studies have linked transition metal oxide and carbon-based active materials to enhance the resistivity and cycle stability of the electrode materials. Here, Wang et al. designed urchin-like MgCo_2_O_4_@PPy/Ni foam core-shell structures using a one-step hydrothermal method [25], while Lee et al. fabricated nanostructured FeOOH/PPy via an electrodeposition process [23].

In the present paper, we report the fabrication of quasi-ionic liquid-based polymer gel electrolyte symmetric SCs based on PPy-coated MnO_2_ nanofibers grown on carbon cloth (CC) (PPy-MnO_2_-CC) for use as the positive and negative electrodes. Following this, two PPy-MnO_2_-CC electrodes were assembled within a urea-LiClO_4_-PVA polymer gel electrolyte to form solid-state SSCs (PPy-MnO_2_-CC SSCs). The MnO_2_ nanofibers were effectively deposited on the CC substrate by applying one-step electrodeposition with a constant potential (0.5 V). The PPy-coated MnO_2_ nanofibers were fabricated via a simple soak and self-polymerization process. The PPy-MnO_2_-CC solid-state SSCs presented a high *C_sp_* (270 F/g) and a wide potential window (2.1 V), with an excellent energy density of up to 165.3 Wh/kg at a power density of 1 kW/kg, as well as exceptional cycle stability and rate performance (<8% loss after 3000 cycles at 5 A/g). These results demonstrated that a simple method is provided for increasing the energy storage capacity by designing the specific formation of the energy storage system. Designing the powerful pseudocapacitive active materials (PPy-coated MnO_2_ nanofibers) with the polymer gel electrolyte (urea-LiClO_4_-PVA) is also discussed.

## 2. Materials and Methods

### 2.1. Materials

Pyrrole monomer (98%), Mn(CH_3_COOH)_2_∙4H_2_O, urea (>95%), LiClO_4_, FeCl_3_, poly(vinyl alcohol) (PVA, Mw 85,000), H_3_PO_4_, HCl, Na_2_SO_4_, and NaCl were obtained from Sigma-Aldrich Chemical Co. (Taufkirchen, Germany) and were used as received without any purification. The CC (WOS 1010) was obtained from CeTech Co. (Taichung, Taiwan).

### 2.2. Preparation of MnO_2_-CC and PPy-CC Electrodes

The CC was cleansed and activated utilizing baking soda cleaner, deionized (DI) water, acetone, HNO_3_ (2 M), and then DI water again. Electrodeposition was used to produce MnO_2_ nanofibers on the CC substrate using an applied constant potential (0.5 V) from the electroplating solution that included the Mn(CH_3_COOH)_2_ (0.5 M). The overall electrodeposited charge was 1 C/cm^2^. The electrodeposition was executed applying the CC as a working electrode, Pt mesh as a counter electrode, and saturated-calomel electrode (SCE) as a reference electrode. Then, the MnO_2_-CC electrodes were achieved. The mass of MnO_2_ was around 0.4 mg/cm^2^.

The CC electrode (~3 cm^2^) was immersed in the pyrrole monomer for 5 min, before being dispensed into a 25-mL solution with FeCl_3_ (2 g) and 0.2 M HCl and maintained at 4 °C for 3 min. Following this, the electrode was removed and cleaned with 0.1 M NaCl and DI water to obtain the final PPy-CC electrodes. The mass of PPy was around 0.6 mg/cm^2^.

### 2.3. Preparation of PPy-MnO_2_-CC Electrodes

The MnO_2_-CC electrode (~3 cm^2^) was directly immersed in the pyrrole monomer (5 min) before being dispensed into the 25-mL solution with FeCl_3_ (2 g) and 0.2 M HCl and kept at 4 °C for 3 min. Following this, the electrode was removed and cleaned with 0.1 M NaCl and DI water to obtain the final PPy-MnO_2_-CC electrodes. The mass of PPy-MnO_2_ was around 1.0 mg/cm^2^.

### 2.4. Preparation of Urea-LiClO_4_-PVA Polymer Gel Electrolytes

The urea-LiClO_4_ quasi-ionic liquid was fabricated using a urea/LiClO_4_ molar ratio of 4:1 [11,18]. The PVA gel was fabricated by dissolving 4 g of PVA in DI water (40 mL) with continuous stirring at 80 °C until the formation of a clear gel. Following this, the polymer gel electrolyte was created by combining the urea-LiClO_4_ (10 g) with the PVA gel (10 g), with the solution heated overnight (110 °C) under churning until the homogeneous gel electrolyte was obtained. The solution was then cooled at room temperature (27 °C) until a clear gel electrolyte was obtained [11,18].

### 2.5. Construction of Solid-State Flexible Symmetric Supercapacitors (SSCs)

The solid-state flexible SSC was constructed using two PPy-MnO_2_-CC electrodes as the positive electrode and negative electrode. Prior to the construction, each PPy-MnO_2_-CC electrode was immersed into the urea-LiClO_4_-PVA polymer gel (2 h). Then, two PPy-MnO_2_-CC electrodes were set together using the urea-LiClO_4_-PVA polymer gel and left overnight to allow for electrolyte solidification. The urea-LiClO_4_-PVA polymer gel acted as both an electrolyte and an ion porous separator in this SSC system. Three modes of SSC were fabricated: a PPy-MnO_2_-CC type SSC with two PPy-MnO_2_-CC electrodes, a MnO_2_-CC type SSC with two MnO_2_-CC electrodes, and a PPy-CC type SSC with two PPy-CC electrodes.

### 2.6. Material Characterization

General morphological analysis of the samples was carried out using a scanning electron microscope (SEM, JEOL JSM-7610F, Tokyo, Japan), while the surface chemical composition of the samples was investigated via X-ray photoemission spectrometry (XPS, BL 09A2 NSRRC beamline, Hsinchu, Taiwan), and the crystal structure was analyzed using the X-ray diffraction technique (XRD, BL12B1 SPring-8, Saitama, Japan). Meanwhile, the sample mass was determined using a microbalance (XP105DR, readability of 0.01 mg, Mettler Toledo, Greifensee, Switzerland).

### 2.7. Electrochemical Measurements

All cyclic voltammetry (CV), galvanostatic charge-discharge (GCD) curves, and cycling stability measurements were obtained using a potentiostat/galvanostat instrument (PGSTAT 128N, Autolab, Utrecht, The Netherlands). The specific capacitances (*C_sp_*, F/g) of the solid-state SSCs obtained from the CV curves were determined according to the following [11,26]:

*C_sp_* = *specific voltammetric charge*/∆V (1)

where ∆V denotes the operating potential range. Here, the specific voltammetric charge (per gram of the active materials contained in two electrodes) was integrated from positive to negative scans of the CV. Both the energy density (E, W h/kg) and the power density (P, W h/kg) were measured according to the following [11,26]:

E = 1/2 *C_sp_* ∆V^2^ (2)

P = E/∆t(3)

where ∆V, ∆t are the operating potential of the SSC and the discharge time.

## 3. Results and Discussion

### 3.1. Morphology and Structure

**Figure 1:**
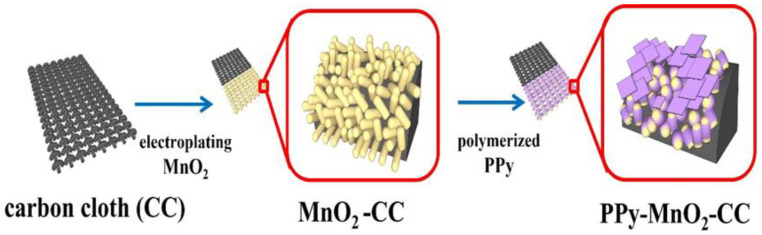
Schematic illustration of the construction process of MnO_2_-CC and PPy-MnO_2_-CC electrodes.

**Figure 2:**
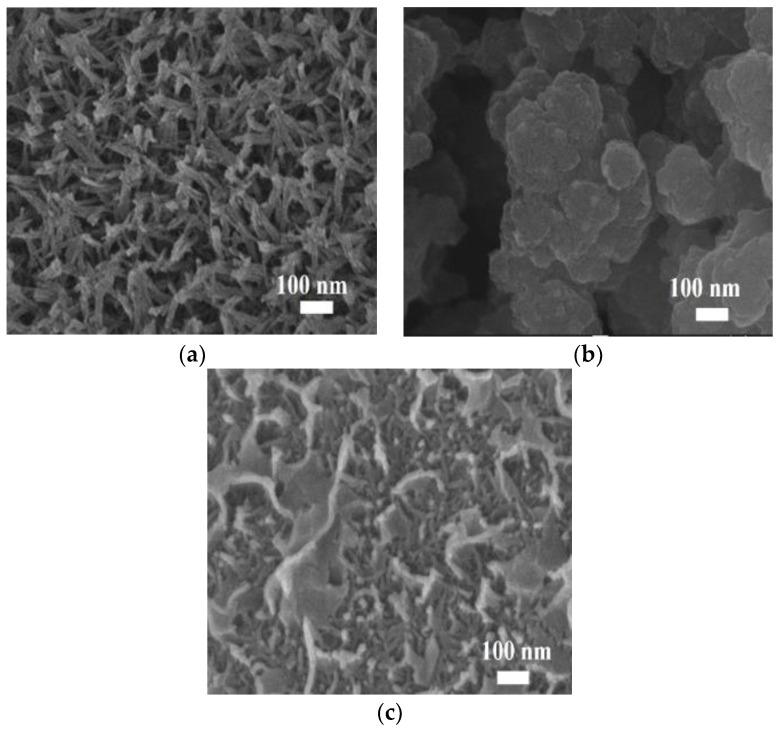
SEM image of (**a**) MnO_2_-CC, (**b**) PPy-CC, and (**c**) PPy-MnO_2_-CC electrodes, respectively.

The construction process for the MnO_2_-CC and PPy-MnO_2_-CC electrodes is displayed in Figure 1 (more details were supplied in the Methods section). The attendant SEM images clearly indicated that vertically oriented MnO_2_ nanofibers (20–30 nm in diameter) and length up to ~1 um was uniformly distributed on the surface of the CC substrate (Figure 2a). Figure 2b shows the SEM image of the PPy-CC electrodes, with the morphology aggregates composed of irregularly shaped particles (100–200 nm). As displayed in Figure 2c, pyrrole monomer was polymerized to PPy onto the surface of the prepared MnO_2_-CC electrode. The prepared MnO_2_/CC electrode was dipped into the FeCl_3_ solution, causing to the Fe^3+^ adsorption onto the MnO_2_ nanofibers and the bare CC. Following the Fe^3+^ adsorption onto the MnO_2_ nanofibers and the bare CC, pyrrole monomer was polymerized to polypyrrole (PPy) through self-polymerization process. The morphology of PPy (Figure 2c) changed into one characterized by smaller nanoflakes and nanoparticles, which matched well with the size of the vertically oriented MnO_2_ nanofibers. Actually, PPy attached to MnO_2_/CC electrode by the bond between the N at the pyrrole rings and -OH groups of MnO_2_ nanofibers by hydrogen bonding. Such a porous and bridge structure offers an extremely open specific surface for promoting the charge transfer and ion diffusion in the electrolyte and thus enhances the rapid redox reaction.

**Figure 3:**
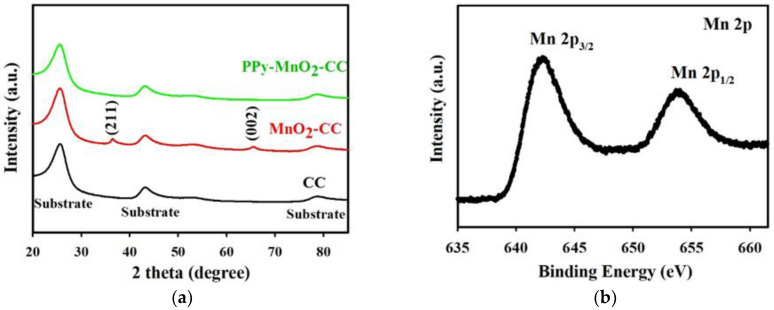

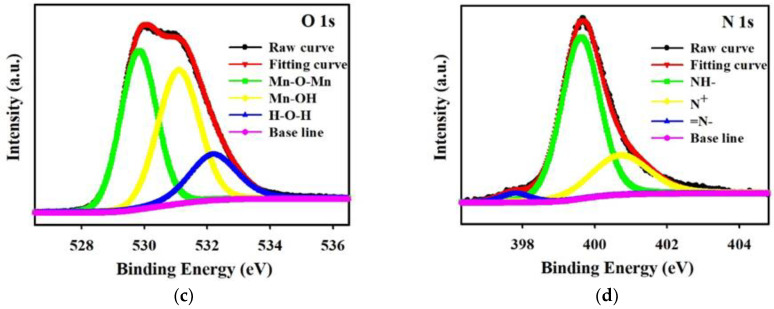
(**a**) XRD patterns of blank CC, MnO_2_-CC, and PPy-MnO_2_-CC electrodes, respectively. XPS spectra of (**b**) Mn 2p, (**c**) O 1s, and (**d**) N 1s of the as-prepared PPy-MnO_2_-CC electrode.

Figure 3a presents the X-ray diffraction (XRD) patterns of the blank CC and the as-prepared MnO_2_-CC and PPy-MnO_2_-CC electrodes. Here, the two smaller diffraction peaks of 37° (211) and 65.7° (002) could be indexed as α-type MnO_2_ (JCPDS 44-0141). Meanwhile, the peak intensity of the PPy-MnO_2_-CC sample was clearly lower than that of the MnO_2_-CC sample, which could be ascribed to the semicrystalline layer of the PPy [23]. Further information on the elemental composition and valence states of the PPy-MnO_2_-CC electrode was obtained via X-ray photoemission spectroscopy (XPS) measurements (Figure 3b,c,d). As shown in Figure 3b, there are two peaks situated at 642.0 eV (Mn 2p_3/2_) and 653.7 eV (Mn 2p_1/2_), which are compatible with those reported in the existing literature for α-type MnO_2_ [27], further verifying the existence of α-MnO_2_ in the PPy-MnO_2_-CC sample. Figure 3c also displays the O 1s spectrum of the PPy-MnO_2_-CC sample, which could be divided into three component peaks relating to the Mn-O-Mn (529.8 eV), the Mn-O-H (531.1 eV) and the adsorbed H_2_O (532.3 eV) [28,29]. The N 1s spectrum was utilized to evaluate the neutral and positive-charged nitrogen in the PPy (Figure 3d). Here, there were three component peaks relating to the deprotonated imine group (=N-) at 397.8 eV, the neutral pyrrolylium nitrogen (-NH-) at 399.6 eV and the charged polaronic nitrogen (N^+^) at 401.2 eV, which also verified that PPy was existed on the as-prepared PPy-MnO_2_-CC electrode [23,30].

### 3.2. Electrochemical Properties of the MnO_2_-CC, PPy-CC, and PPy-MnO_2_-CC Electrodes in a Three-Electrode Cell with Different Electrolytes

**Figure 4:**
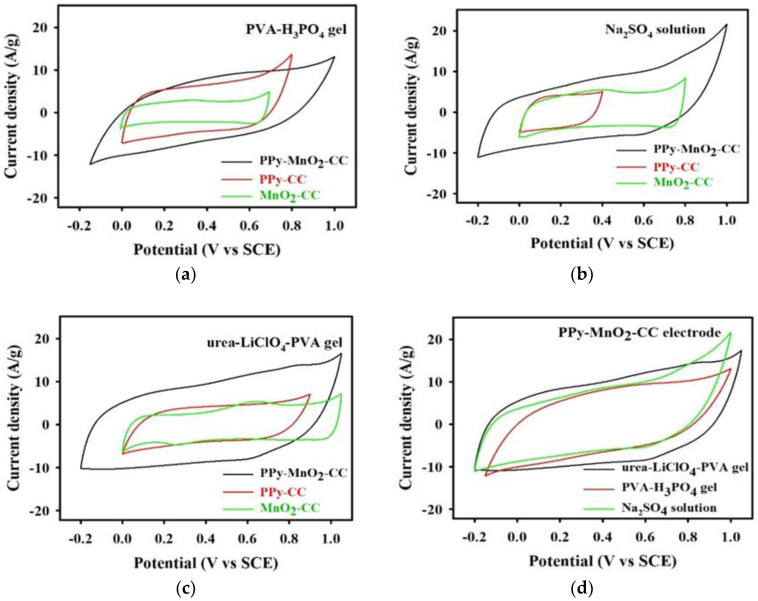
(**a**) CV curves of the PPy-MnO_2_-CC, PPy-CC, and MnO_2_-CC electrodes recorded with 1 M Na_2_SO_4_ (10 mV/s). (**b**) CV curve of the PPy-MnO_2_-CC, PPy-CC, and MnO_2_-CC electrodes recorded with PVA-H_3_PO_4_ gel electrolyte (10 mV/s). (**c**) CV curve of the PPy-MnO_2_-CC, PPy-CC, and MnO_2_-CC electrodes recorded with urea-LiClO_4_-PVA gel electrolyte (10 mV/s). (**d**) CV curves of the PPy-MnO_2_-CC electrode recorded with different electrolytes (urea-LiClO_4_-PVA gel, PVA/H_3_PO_4_ gel, and Na_2_SO_4_ solution).

To estimate the electrochemical performance of the active material electrodes in relation to energy storage device application, the electrochemical properties were examined in a 1 M Na_2_SO_4_ solution, PVA-H_3_PO_4_ gel and urea-LiClO_4_-PVA gel as the electrolytes applying a three-electrode cell, respectively. Figure 4a shows the voltammograms of the MnO_2_-CC, PPy-CC, and PPy-MnO_2_-CC electrodes, as measured in the 1 M Na_2_SO_4_ (10 mV/s). All the CV curves were essentially rectangular shaped with the different enclosed areas and working potential ranges. As Figure 4a shows, the PPy-MnO_2_-CC electrode can be measured in a potential range of 1.2 V (−0.2 V to +1.0 V), which is larger than the PPy-CC electrode of 0.4 V (0 V to +0.4 V) and MnO_2_-CC electrode of 0.8 V (0 V to +0.8 V). It was observed that the PPy-CC electrode was obviously dissolved 0.1 M Na_2_SO_4_ solution as the CV scan range over 0.4V. The PPy-MnO_2_-CC electrode clearly exhibited the best enclosed area, indicating the ideal pseudocapacitive behavior of the PPy-MnO_2_-CC electrodes in the 1 M Na_2_SO_4_ solution. The specific capacitance (*C_sp_*) of the PPy-MnO_2_-CC, PPy-CC and MnO_2_-CC electrodes are 601, 323, and 357 F/g, respectively. As found from Figure 4a, the CV curves of PPy-MnO_2_-CC electrode demonstrated the remarkable *C_sp_* compared to the PPy-CC and MnO_2_-CC electrodes, which can be ascribed to the capacitance enhancement from the electrochemical doping/updoping process of SO_4_^−2^ of the PPy from the nanostructured PPy-coated MnO_2_ composite electrode during charging/discharging process [23], and better MnO_2_ nanofibers application through PPy enhanced charge transfer.

Meanwhile, Figure 4b displays the voltammograms of the three electrodes, as measured in the PVA-H_3_PO_4_ gel electrolyte (10 mV/s). Here, both the current density and the working potential of PPy-MnO_2_-CC electrode were wider than those of the PPy-CC and MnO_2_-CC electrode. The PPy-MnO_2_-CC electrode can be determined in a potential range of 1.1 V (−0.1 V to +1.0 V), which is also greater than the PPy-CC electrode of 0.8 V (0 V to +0.8 V) and MnO_2_-CC electrode of 0.7 V (0 V to +0.7 V). It was noted that the MnO_2_-CC electrode was also slowly dissolved in the PVA-H_3_PO_4_ gel electrolyte as the CV scan range over 0.7 V. The *C_sp_* of the PPy-MnO_2_-CC, PPy-CC, and MnO_2_-CC electrodes are 558, 504, and 247 F/g, respectively. Furthermore, the electrochemical behavior of the three electrodes was measured in the urea-LiClO_4_-PVA gel using the CV curves (10 mV/s), as shown in Figure 4c, with the PPy-MnO_2_-CC electrode clearly presents the most prospective performance among the three electrodes. The PPy-MnO_2_-CC electrode can be measured in a potential range of 1.2 V (−0.2 V to +1.0 V). The *C_sp_* of the PPy-MnO_2_-CC, PPy-CC, and MnO_2_-CC electrodes are 652, 352, and 383 F/g, respectively. Thus, the PPy-MnO_2_-CC electrode could both improved the working potential window and pseudocapacitive performance compared to other electrodes (Figure 4c). Figure 4d also displays the CV curves of the PPy-MnO_2_-CC electrode in urea-LiClO_4_-PVA gel, the PVA-H_3_PO_4_ gel, and the Na_2_SO_4_ solution, respectively. Here, the CV curve for the PPy-MnO_2_-CC electrode in the urea-LiClO_4_-PVA gel presented a largely rectangular-like shape, indicating ideal pseudocapacitive behavior, while it also exhibited the largest electrochemically active area, indicating that it had the highest *C_sp_* (652 F/g) among all the electrolytes. These remarkable electrochemical properties indicate that the PPy-MnO_2_-CC electrode can directly applied for pseudocapacitor electrode. Here, it should be noted that the urea-LiClO_4_-PVA gel electrolyte can be used to replace the traditional acid gel polymer electrolytes.

### 3.3. Electrochemical Performance Testing for the PPy-MnO_2_-CC, PPy-CC, and MnO_2_-CC Solid-State Flexible Symmetric Supercapacitors

**Figure 5:**
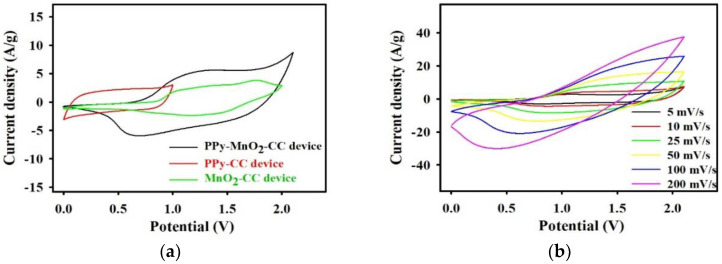

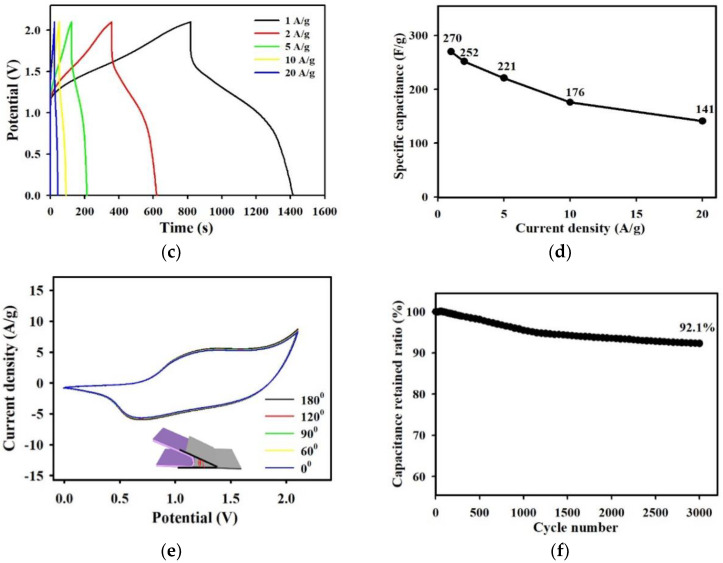
(**a**) CV curves of the PPy-MnO_2_-CC, PPy-CC and MnO_2_-CC flexible SSC recorded with the urea-LiClO_4_-PVA gel electrolyte (10 mv/s). (**b**) CV curve of the PPy-MnO_2_-CC flexible SSC device at various scan rates. (**c**) GCD curves of the flexible SSC device at various current densities (1–20 A/g). (**d**) *C_sp_* of the flexible SSC device as a function of current density. (**e**) CV curves of the flexible SSC device at different bending states (10 mV/s). (**f**) Cycle stability of the flexible SSC device at 5 A/g.

To further study the capacity of the PPy-MnO_2_-CC electrode for electrochemical energy storage, a quasi-solid-state flexible SSC device was constructed utilizing the PPy-MnO_2_-CC electrode as both electrodes, lens cleaning paper as separator, and the urea-LiClO_4_-PVA gel as electrolyte. Here, PPy-CC and MnO_2_-CC type SSC devices were constructed for comparisons. Figure 5a displays the CV curves for the PPy-MnO_2_-CC, PPy-CC, and MnO_2_-CC flexible SSCs manufactured using the urea-LiClO_4_-PVA gel electrolyte, with the PPy-MnO_2_-CC flexible SSC device presenting a large electrochemical area. The apparent redox peaks in the CV curve of the PPy-MnO_2_-CC flexible SSC device suggested common pseudocapacitive properties of MnO_2_ and PPy in the urea-LiClO_4_-PVA gel electrolyte, which significantly enhanced the *C_sp_*. From Figure 5a, the PPy-MnO_2_-CC SSC devices can be measured in a potential range of 2.1V, which is larger than the PPy-CC electrode of 1.0 V and MnO_2_-CC electrode of 2.0 V. The specific capacitance (*C_sp_*) of the PPy-MnO_2_-CC, PPy-CC, and MnO_2_-CC electrodes are 270, 104, and 100 F/g, respectively. The CV curves of PPy-MnO_2_-CC flexible SSC device demonstrated the remarkable *C_sp_* compared to the PPy-CC and MnO_2_-CC SSC devices, which can be ascribed to the capacitance enhancement from the electrochemical doping/updoping process of ClO_4_^−^ of the PPy from the nanostructured PPy-coated MnO_2_ composite electrode during charging/discharging process [23], and better MnO_2_ nanofibers application through PPy improved charge transfer. This results also indicated that the PPy-MnO_2_-CC type flexible SSC with the urea-LiClO_4_-PVA gel electrolyte could significantly widen its working potential window (2.1V) without lost too much capacity. Furthermore, the PPy-MnO_2_-CC flexible SSC demonstrated excellent electrochemical behavior, even at 200 mV/s (55.6% retention estimated at 5 mV/s), exhibiting a rapid charge–discharge property for power systems (Figure 5b). A pair of redox peaks on the CVs was noticed which should be associated with the faradaic redox reactions. The peak current densities enhanced with increasing scan rates, but *C_sp_* values of the PPy-MnO_2_-CC flexible SSC determined from the enclosed area of the CVs were not significantly affected, showing the excellent rate capability. Meanwhile, Figure 5c shows the GCD curves for the PPy-MnO_2_-CC type flexible SSC from 0 to 2.1 V at various current densities. Here, the GCD curves were not completely linear, which was consistent with the redox peaks (refer to CV curves in Figure 5b, showing a pseudocapacitive property with a slope change in the time dependence of the potential, indicating the insertion/extraction of cations from urea-LiClO_4_-PVA gel electrolyte. [23,31]. Figure 5d displays the *C_sp_* as determined from the GCD curves as a function of the current density, with the *C_sp_* potentially obtained by the PPy-MnO_2_-CC flexible SSC device found to be 270, 252, 221, 176, and 141 F/g at 1, 2, 5, 10, and 20 A/g, respectively (Figure 5d). Figure S1 displays the capacitance retention of the PPy-MnO_2_-CC and MnO_2_-CC flexible SSC devices after revealing to various current densities; this also indicates that the *C_sp_* recover after high current densities. As the current density is decreased from 20 to 5 and 1 A /g the *C_sp_* recovers to the values of 64.2% and 98.4% (for the PPy-MnO_2_-CC SSC device), respectively. By contrast, for the MnO_2_-CC SSC device, 51.7% and 89.8%, respectively, of the capacitance was maintained. The PPy-MnO_2_-CC flexible SSC device has high capacitance retention after obtaining the high current density (20 A/g) to be ascribed to two key factors: (1) the PPy in the composite electrode could enhances the urea-LiClO_4_-PVA polymer gel achievability that is valuable to the rapid redox reaction; (2) the novel conducting networks decrease the ion/electron diffusion pathways across the active material, improving the electrode conduction for greater reaction kinetics. A maximum energy density of 165.3 Wh/kg was carried at a power density of 1.0 kW/kg according to Equations (2) and (3), which is better than that of the majority of previously reported SSCs, including PPy-coated carbon nanotube/cotton hybrid fabric SSCs (12.6 Wh/kg, 95% after 5000 cycles) [32], agarose-bound activated carbons SSCs (13.5 Wh/kg, 94.2% after 10,000 cycles) [26], self-assembled graphene foam (13.5 Wh/kg, 81% after 3000 cycles) [33], V_2_O_5_-PPy composite/CC//V_2_O_5_-PPy composite/CC SSC (82.0 Wh/kg, 75% after 5000 cycles) [34], V_2_O_5_-PANI composite//V_2_O_5_-PANI composite SSCs (69.2 Wh/kg, 92% after 5000 cycles) [35], activated CF//activated CF SSCs (4.0 Wh/kg, 80% after 10,000 cycles) [36], PPy/black phosphorus SSCs (30.8 Wh/kg, 95% after 10,000 cycles) [37], and MnO_2_@PANI/GF// MnO_2_@PANI/GF SSCs (37.0 Wh/kg, 89% after 5000 cycles) [38]. Meanwhile, we also achieved a maximum power density of 21.0 kW/kg at a reasonable energy density of 86.4 W h/kg. 

**Figure 6:**
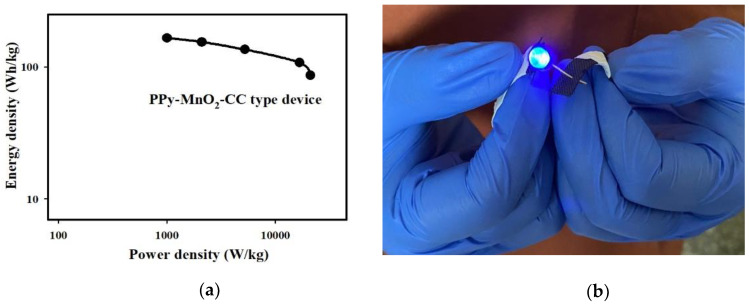
(**a**) Ragone plots of the PPy-MnO_2_-CC flexible SSC device. (**b**) Blue LED operated by a single device.

Table 1 presents comparisons between the electrochemical performance of the PPy-MnO_2_-CC flexible SSC devices and that of previously reported flexible SCs. The symmetric SC devices is limited in the previous reports. Most of the research papers tried to combine transition metal oxide and conductive polymers (or carbon materials) such as V_2_O_5_-PPy, V_2_O_5_-PANI, MnO_2_@PANI, FeCo_2_O_4_@PPy, VO_2_/CNT, CC/CW/Fe_3_O_4_@C, and MnO_2_/CF//Graphene composite materials to enhance the energy storage capability of the transition metal oxide-based device (see Table 1). However, the potential window of the SSC devices using LiCl/PVA or 1 M Na_2_SO_4_(aq) as electrolyte is usually smaller than those using urea-LiClO_4_-PVA electrolyte. In comparison with various composite electrode materials and electrolytes, the energy density of PPy-MnO_2_-CC flexible SSCs with urea-LiClO_4_-PVA electrolyte is significantly higher than those of SSCs (see Table 1). The CV curves of the PPy-MnO_2_-CC flexible SSC attained at 10 mV/s remained almost unchanged under different bending angles (Figure 5e), demonstrating its potential for flexible/wearable energy storage systems. Furthermore, as Figure 5f shows, the PPy-MnO_2_-CC flexible SSC retained outstanding cycle stability, with a 92.1% capacitance retention after 3000 cycles (5 A/g). To further identify the electrochemical cycle stability and durability of the PPy-MnO_2_-CC flexible SSC device, the device under different geometrical states was performed by CP at 5 A/g (Figure S2). Moreover, 5000 cycles, involving folding, bending, and twisting in the range of 0–2.1 V and the results appear that the PPy-MnO_2_-CC flexible SSC device kept 86.5% of the original capacitance. All these investigations supported the conclusion that the PPy-MnO_2_-CC flexible SSC device possessed excellent cyclic stability and rate capability. Meanwhile, the Ragone plot of the PPy-MnO_2_-CC flexible SSC device is shown in Figure 6a, while the blue light-emitting diode (LED) operated by the PPy-MnO_2_-CC type flexible SSC device is shown in Figure 6b.

The outstanding pseudocapacitive performance of the examined 2.1 V PPy-MnO_2_-CC flexible SSCs was fully demonstrated (Figure 5) and could be attributed to the nanostructured PPy-coated MnO_2_ electrode and the urea-LiClO_4_-PVA polymer gel electrolyte design. The design of such electrode structure and polymer gel electrolyte exhibits the following aims: First, the MnO_2_ nanofibers were applied as the porous self-supporting substrates to load more PPy active materials in view of significantly reducing the “dead space” and improving the utilization of active materials and subsequently producing a higher *C_sp_* and rate capability. Second, the PPy nanoflake and nanoparticle morphologies also provided the electrodes with a large surface area and fast redox reactions (from the electrochemical doping/updoping process of ClO_4_^−^ of the PPy from the nanostructured PPy-MnO_2_-CC composite electrode), resulting in superior capacity and an outstanding rate capability and expressway for rapid charge storage and electron transfer. Third, the urea-LiClO_4_-PVA polymer gel electrolyte not only enhanced the conductivity and buffered the volume change but also improved the working potential window and insertion/extraction of cations from urea-LiClO_4_-PVA gel electrolyte. Overall, the efficient design of the electrode and electrolyte with high potential windows and a large specific capacity resulted in SSC devices with a high operating voltage and excellent energy/power densities.

## 4. Conclusions

In this paper, we fabricated a MnO_2_ nanofiber self-supporting substrate coated with PPy nanomaterials growth on a CC substrate (PPy-MnO_2_-CC) to serve as the electrodes subsequently linked with a urea-LiClO_4_-PVA polymer gel electrolyte for flexible SSCs. The PPy-MnO_2_-CC flexible SSC device demonstrated a wide operating voltage from 0 to 2.1 V and a significantly improved *C_sp_* (270 F/g at 1 A/g). Furthermore, owing to the wide working potential and excellent *C_sp_*, the PPy-MnO_2_-CC flexible SSC device presented an outstanding energy density of 165.3 W h/kg, as well as excellent cyclic stability (capacitance retention of 92.1% retention after 3000 cycles) and rate capability (141 F/g at 20 A/g), outperforming the majority of previously reported flexible SCs. We also achieved a maximum power density of 21.0 kW/kg at a reasonable energy density of 86.4 W h/kg. The outstanding pseudocapacitive performance of the examined 2.1 V PPy-MnO_2_-CC flexible SSCs could be attributed to the nanostructured PPy-coated MnO_2_ electrode and the urea-LiClO_4_-PVA polymer gel electrolyte design. Overall, this work presents a simple pathway to designing flexible SCs with high voltage and excellent performance.

## Figures and Tables

**Table 1:** Comparison of flexible supercapacitors.

Electrode Material	Electrolyte	Potential Windows (V)	Energy Density (Wh/Kg)	Ref.
PPy-coated CNT/cotton hybrid fabric	H_2_SO_4_/PVA	0.8	12.6	[32]
CC/CW/Fe_3_O_4_@C// CC/CW/MnO_2_	1 M Na_2_SO_4(aq)_	2.6	91.1	[12]
V_2_O_5_-PPy/CC// V_2_O_5_-PPy/CC	LiCl/PVA	2.0	82.0	[34]
V_2_O_5_-PANI// V_2_O_5_-PANI	LiCl/PVA	1.8	69.2	[35]
activated CF// activated CF	EMIMBF_4_-Li_2_SO_4_-Agar/PVA	1.0	4.0	[36]
FeCo_2_O_4_@PPy//PPy @VO_2_/CNT	LiCl/PVA	1.8	68.8	[39]
PPy/Black Phosphorus	H_3_PO_4_/PVA	0.6	30.8	[37]
MnO_2_/CF//Graphene/ MnO_2_@CNT	LiCl/PVA	1.5	27.2	[40]
MnO_2_@PANI/GF// MnO_2_@PANI/GF	KOH/PVA	1.5	37.0	[38]
PPy-MnO_2_-CC// PPy-MnO_2_-CC	1 M Na_2_SO_4(aq)_	1.4	57.1	This work
PPy-MnO_2_-CC// PPy-MnO_2_-CC	LiCl/PVA	1.8	65.3	This work
MnO_2_-CC// MnO_2_-CC	urea-LiClO_4_-PVA	2.0	55.6	This work
PPy-MnO_2_-CC// PPy-MnO_2_-CC	urea-LiClO_4_-PVA	2.1	165.3	This work

## Data Availability

Data is contained within the article.

## Supplementary Materials

The following are available online at https://www.mdpi.com/article/10.3390/polym13203577/s1, Figures S1–S2: Enhanced Pseudocapacitive Performance of Symmetric Polypyrrole-MnO2 Electrode and Polymer Gel Electrolyte.
