# Supplementary material for: Enhanced Pseudocapacitive Performance of Symmetric Polypyrrole-MnO2 Electrode and Polymer Gel Electrolyte
Source: Polymers (Basel). 2021 Oct 16;13(20):3577. doi: 10.3390/polym13203577 (PMC8539299; doi:10.3390/polym13203577)
Supplement: Supplementary file 1 [file polymers-13-03577-s001.zip › polymers-1417904-supplementary.pdf]

Supplementary Materials

Enhanced Pseudocapacitive Performance of Symmetric Polypyrrole-MnO_2 Electrode and Polymer Gel Electrolyte

Wen-Jun Zhuo[1], Yen-Hua Wang[1], Chia-Tse Huang[1], Ming-Jay Deng[1]*

Department of Applied Chemistry, Providence University, Taichung 43301, Taiwan

* Correspondence: dengmj1020@pu.edu.tw (M.J. Deng)

[1] These authors contributed equally to this work.

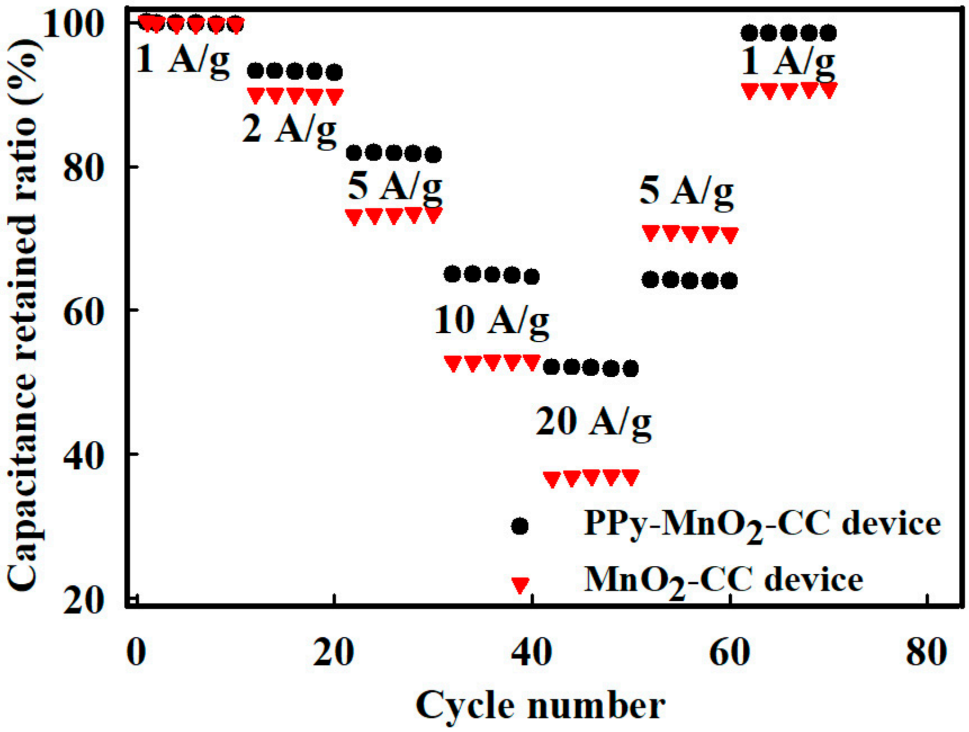

Figure S1 Rate performance tests of the PPy-MnO_2-CC and MnO_2-CC flexible SSC devices.

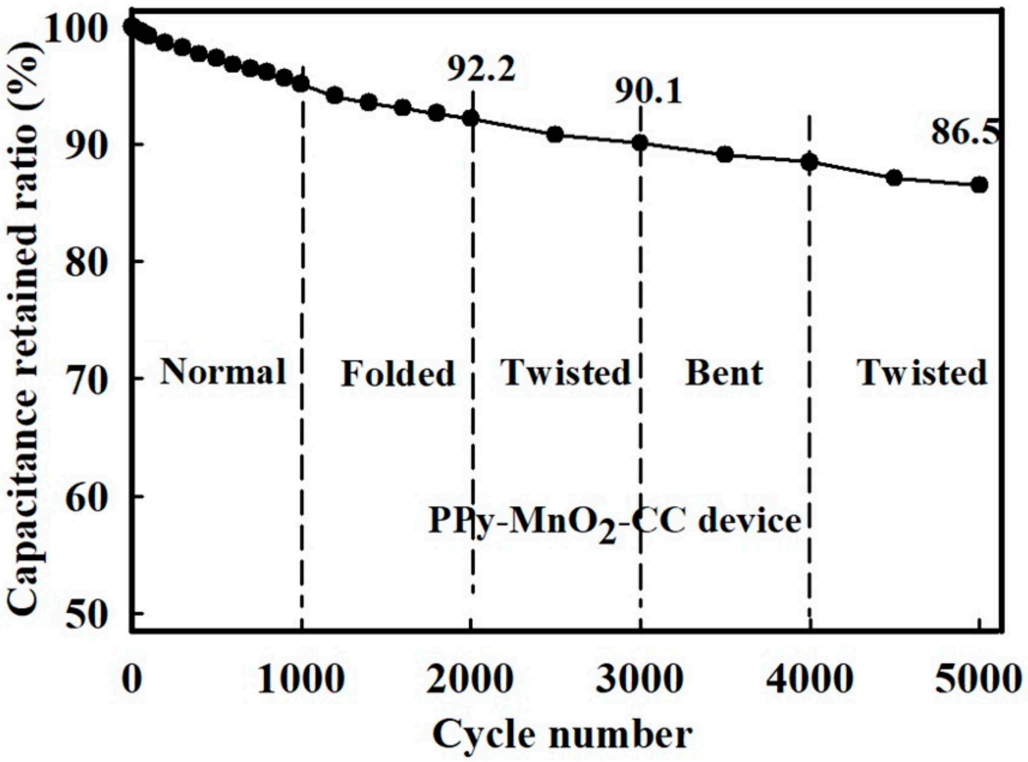

Figure S2 Cycle stability and durability of the PPy-MnO_2-CC flexible SSC device under various bending and twisting test conditions by CP at 5 A/g.
